# Organization of lipids in milks, infant milk formulas and various dairy products: role of technological processes and potential impacts

**DOI:** 10.1007/s13594-015-0263-0

**Published:** 2015-11-02

**Authors:** Christelle Lopez, Chantal Cauty, Fanny Guyomarc’h

**Affiliations:** INRA, UMR1253 STLO, 65 rue de Saint Brieuc, 35000 Rennes, France; Agrocampus Ouest, UMR1253 STLO, 65 rue de Saint Brieuc, 35000 Rennes, France

**Keywords:** Milk, Fat, Milk fat globule membrane, Homogenization, Structure

## Abstract

The microstructure of milk fat in processed dairy products is poorly known despite its importance in their functional, sensorial and nutritional properties. However, for the last 10 years, several research groups including our laboratory have significantly contributed to increasing knowledge on the organization of lipids in situ in dairy products. This paper provides an overview of recent advances on the organization of lipids in the milk fat globule membrane using microscopy techniques (mainly confocal microscopy and atomic force microscopy). Also, this overview brings structural information about the organization of lipids in situ in commercialized milks, infant milk formulas and various dairy products (cream, butter, buttermilk, butter serum and cheeses). The main mechanical treatment used in the dairy industry, homogenization, decreases the size of milk fat globules, changes the architecture (composition and organization) of the fat/water interface and affects the interactions between lipid droplets and the protein network (concept of inert vs active fillers). The potential impacts of the organization of lipids and of the alteration of the milk fat globule membrane are discussed, and technological strategies are proposed, in priority to design biomimetic lipid droplets in infant milk formulas.

## Introduction

Milk is a biological fluid secreted by all female mammals for the growth and development of newborns. Directly transferred from the mother to the newborn, all the constituents of milk (e.g. proteins, lipids, lactose and oligosaccharides) are in their natural state and adapted to bring energy and bioactive molecules. Milk from animals (e.g. cows, buffaloes and goats) is also transformed into various dairy products for infant and adult human consumption.

In particular, milk lipids are secreted in the form of colloidal assemblies called milk fat globules. Technological processes used in the dairy industry, e.g. thermal and mechanical (pumping and homogenization) treatments, can affect the structure and composition of milk components and alter their technological and nutritional properties (Michalski et al. 2013; Garcia et al. 2014). Hence, it is important to have a good knowledge about the organization of lipids in dairy products. The recent development of microscopy techniques has permitted structural investigations in situ in complex food products.

The objectives of this paper are to update information about the organization of lipids in unprocessed milk and to show the role of technological processes on the organization of lipids in processed milk, infant formulas and various dairy products (e.g. cream, butter, buttermilk and cheeses). The major role played by microscopy techniques for the characterization of lipids in situ in milk and processed dairy products is highlighted. This overview compiles the most recent research advances in the field of the organization of milk lipids and brings original structural data in complex dairy products. The potential impacts of the organization of lipids in processed dairy products as compared to unprocessed milk fat globules on the digestibility and metabolic fate of milk lipids are discussed.

For the last 10 years, our laboratory UMR 1253 Science and Technology of Milk and Eggs from the French National Institute for Agricultural Research (INRA) has contributed significantly to the advancement of knowledge on the organization of lipids in the milk fat globule membrane and in situ in various dairy products. Our expertise and research approaches are highlighted throughout the overview.

## Materials and methods

### Milks, infant milk formulas and processed dairy products

Unprocessed whole bovine milks were provided by a local dairy plant (Triballat, Noyal sur Vilaine, France). Mature human milks were donated by volunteers, as described by Lopez and Ménard (2011). Sterilized UHT, pasteurized and microfiltered fluid milks were bought in supermarkets. Infant milk formulas for 0–6-month babies were provided by industrials or bought in supermarkets (Rennes, France). Sour creams, UHT creams and liquid creams “crème fleurette” were bought in local supermarkets (Rennes, France). Industrial butter, buttermilks and butter serum were provided by a local dairy company (Brittany, France). The cheeses were provided by dairy companies, bought in local supermarkets (Rennes, France) or manufactured in the INRA-STLO pilot plant Plateforme Lait (Lopez et al. 2008).

### Particle size measurements

The size distributions of milk fat globules and processed lipid droplets were determined by laser light scattering using a Mastersizer 2000 (Malvern Instruments, UK) with two laser sources. The refractive indexes used for the laser at 633 and 466 nm were 1.458 and 1.460 for milk fat and 1.462 and 1.460 for blends of vegetable oils in infant formulas, respectively. The refractive index of 1.333 was used for water. About 0.2 mL of sample was diluted in 100 mL of water directly in the measurement cell of the apparatus in order to reach 10% obscuration. The casein micelles were dissociated by adding 1 mL of 35 mM ethylenediaminetetraacetic acid (EDTA)/NaOH, pH 7 buffer to the milks, directly in the apparatus. To determine the size distribution of fat globules in sour creams, samples were diluted 1:10 *v*/*v* in 1% sodium dodecyl sulfate (SDS) in order to dissociate lipid droplet aggregates and the casein network. Standard parameters were calculated by the software. The experiments were performed in triplicate for each sample.

### Confocal microscopy

The microstructural analysis of the milks, infant milk formulas and dairy products (i.e. creams, buttermilk and cheeses) was performed with an inverted microscope NIKON Eclipse-TE2000-C1si (NIKON, Champigny sur Marne, France) allowing confocal laser scanning microscopy (CLSM) and optical microscopy using differential interferential contrast (DIC, also called Nomarski contrast). Confocal experiments were performed using an argon laser operating at 488 nm excitation wavelength (emission was detected between 500 and 530 nm), a He–Ne laser operating at 543 nm wavelength excitation (emission was detected between 565 and 615 nm) and a diode operating at 633 nm detected with a long pass filter >650 nm. The observations were performed using ×40 (NA 1.33) and ×100 (NA 1.4) oil immersion objectives.

Apolar lipids (i.e. triacylglycerols) were stained either with the lipid-soluble Nile Red fluorescent dye (5H-Benzo α phenoxazine-5-one, 9-diethylamino; λex = 543 nm; Sigma-Aldrich, St Louis, USA) or with LipidTox® (λex = 488 nm; Invitrogen). Milk phospholipids were stained with *N*-(lissamine rhodamine B sulfonyl) dioleoylphosphatidyl ethanolamine (Rhodamine-DOPE, Avanti Polar Lipids Inc., Birmingham, UK) provided at a concentration of 1 mg.mL^-1^ in chloroform (chloroform was evaporated under nitrogen to avoid the possible artefacts caused by this organic solvent). The carbohydrate moities were localized in the milk fat globule membrane by using the lectin wheat germ agglutinin Alexa fluor 488 (WGA; Invitrogen, Cergy Pontoise, France). The staining of proteins was performed by using Fast Green FCF (Sigma-Aldrich, St. Louis, USA) prepared at a concentration of 10 mg.mL^-1^ in water. Acridine Orange fluorescent dye (Aldrich Chemical Company, Inc., Milwaukee, USA) was used to label simultaneously the proteins and the bacteria. The staining protocols followed previously described methods to investigate the microstructure of fat globules in situ in milk (Lopez et al. 2010; Lopez and Ménard 2011), the microstructure of cheeses (Lopez et al. 2007, 2008) and the microstructure of milks during in vitro digestion (Garcia et al. 2014). The two-dimensional images had a resolution of 512 × 512 pixels, and the pixel scale values were converted into micrometers using a scaling factor. In the multiple labeled samples, different colours were used to locate the fluorescent probes (e.g. red colour for phospholipids stained by Rh-DOPE or triacylglycerols stained by Nile red, green colour for proteins stained by Fast green FCF or the carbohydrate moieties stained by WGA-488 and grey levels for DIC images).

### Atomic force microscopy

The preparation of supported lipid bilayers of milk sphingomyelin (MSM), dioleoylphosphatidylcholine (DOPC) and/or cholesterol (chol) has been previously detailed by Guyomarc’h et al. (2014). The preparation of Langmuir-Blodgett monolayers has been detailed by Murthy et al. (2015). AFM imaging of the bilayers and Langmuir-Blodgett monolayers was performed in contact mode using an MFP-3D Bio AFM (Asylum Research, Santa Barbara, CA, USA), silicon MSNL probes (nominal spring constant *k* ∼0.03 N.m^-1^, Bruker Nano Surfaces, Santa Barbara, CA, USA) calibrated using the thermal noise method and loading forces below ∼1 nN. Temperature was 23 ± 0.7°C. The typical scan rate was 0.5 Hz for 256 × 256 pixel images. Sections were drawn across images to measure the height difference *H* between features of the images (i.e. difference in height between the domains and the fluid phase). Force mapping was performed on MSM/DOPC (50:50 mol%) and MSM/DOPC/cholesterol (40:40:20 mol%) bilayers as described by Guyomarc’h et al. (2014).

### Transmission electron microscopy

For transmission electron microscopy (TEM) analysis, the industrial buttermilk and butter serum samples were concentrated 5-fold at 45°C using ultrafiltration with a scale-laboratory pilot (Pall Centramate) equipped with polymeric membrane (Pall Omega medium screem, cutoff 10 kDa). Then, the concentrated samples were diluted 1:2 *v*/*v* in agar, fixed for 3 h at room temperature in a solution with final concentration 2.5% glutaraldehyde in 0.15 M Na cacodylate pH 7.2, followed by the post-fixation for 1 h at room temperature in a solution with final concentration 1% osmium in 0.15 M Na cacodylate at pH 7.2. After each fixation, the samples were washed in 0.15 M Na cacodylate at pH 7.2. Samples were dehydrated through an ascending series of ethanol concentrations, infiltrated and embedded in Epon-Araldite-DMP30 resin mixture, then finally polymerized at 60°C for 24 h. Ultra-thin sections (90 nm) were cut in a Leica ultra-microtome and stained with 4% uranyl acetate for 1 h. Sections were investigated with a JEOL 1400 TEM operating at 120 kV; images were recorded on camera Gatan Orius SC 1000 at the Microscopy Rennes Imaging Center (MRic, University Rennes 1, France).

## Overview of the organization of lipids in unprocessed milk fat globules and changes induced by industrial technology: focus on the interface

### Unprocessed milk fat globules: a core of triacylglycerols covered by a biological membrane

**Fig. 1:**
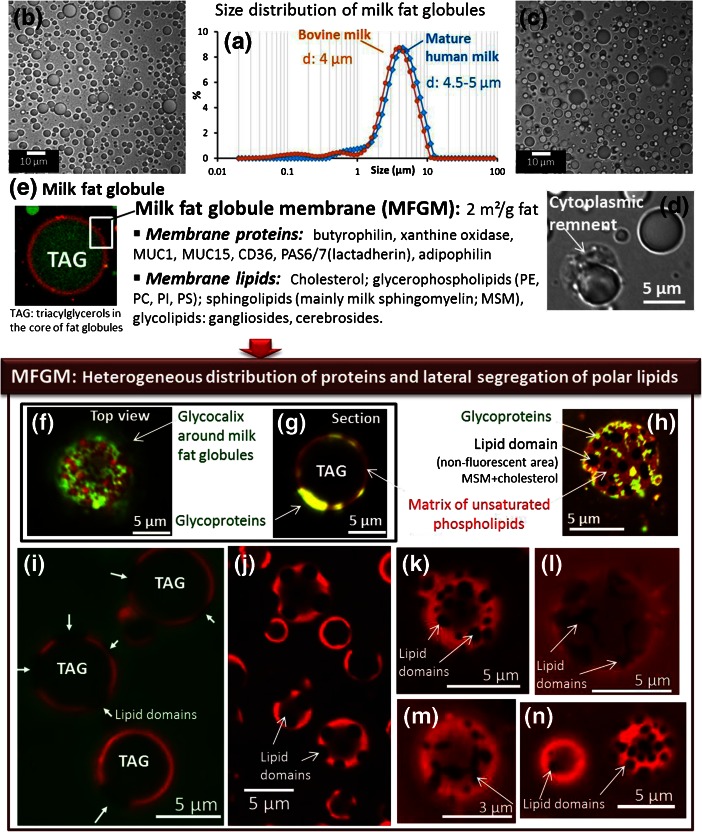
Microstructure of milk fat globules: size distribution and organization of proteins and polar lipids in the milk fat globule membrane (MFGM). Size distributions of milk fat globules in bovine and human milks, determined **a** by laser light scattering and **b, c** microscopy, **d** cytoplasmic remnant attached to a human milk fat globule, **e** confocal laser scanning microscopy (CLSM) image showing the triacylglycerol core (TAG; Lipidtox fluorescent dye, *green colour*) and the biological membrane enveloping milk fat globules (Rhodamine-DOPE fluorescent dye, *red colour*). **f-n** CLSM images showing **f**–**h** the heterogeneous distribution of proteins and glycoproteins in the MFGM, and **h**–**n** the heterogeneous distribution of polar lipids in the outer bilayer of the MFGM (Rhodamine-DOPE fluorescent dye, *red colour*) with the formation of lipid domain highlighted by *arrows* (non-fluorescent areas)

Lipids are secreted in milk in the form of spherical entities of about 4 to 5 μm diameter called milk fat globules (Fig. 1a–c), which are enveloped by a biological membrane known as the milk fat globule membrane (MFGM; Fig. 1d). These colloidal assemblies are natural vehicles for triacylglycerols (TAG: ∼98% *w*/*w* of total milk lipids), fat-soluble nutrients (e.g. carotenoids and vitamins) and other biologically active molecules (e.g. phospholipids, sphingolipids, gangliosides, cholesterol and MFGM proteins). Cytoplasmic remnants from the mammary cells were characterized around milk fat globules, particularly in human milk (Fig. 1e; Lopez and Ménard 2011). The amount of fat in the milk, the size of milk fat globules and the chemical composition of milk lipids, both TAG and polar lipids, are sensitive to the diet (Lopez et al. 2014). Several reviews on milk fat globules have been recently published (Lopez et al. 2011; Gallier et al. 2014). In this part of the overview dedicated to unprocessed milk fat globules, we will focus on the most recent advances about the organization of the MFGM obtained by microscopy techniques. The last 10 years have seen an expanding interest in the structure of the MFGM, largely due to its important roles in the physical stability and techno-functional properties of milk fat globules, as well as in the mechanisms of milk fat digestion and metabolism. The MFGM is composed of polar lipids (phosphatidylcholine, PC; phosphatidylethanolamine, PE; phosphatidylinositol, PI; phosphatidylserine, PS and milk sphingomyelin, MSM), gangliosides, cholesterol, proteins and glycoproteins (Dewettinck et al. 2008; Lopez 2011; Gallier et al. 2014). Organization of the MFGM as a trilayer structure is now well accepted (thickness: 10 to 20 nm), with the inner layer composed of proteins and polar lipids from the endoplasmic reticulum and the outer bilayer of polar lipids originating from specialized secretory regions of the apical plasma membrane of the mammary epithelial cells (Heid and Keenan 2005). The distribution of MFGM polar lipids is asymmetric, with PC and MSM largely located in the outer layer of membrane and PE, PI and PS concentrated in the inner surface Deeth (1997). By using freeze-fracture immunochemistry, Robenek et al. (2006) performed an excellent work that has improved knowledge about the location of proteins in the MFGM. The main research advances regarding the heterogeneities in the outer bilayer of the MFGM have been provided by using confocal microscopy and atomic force microscopy.

#### Lateral segregation of lipids with phase coexistence and heterogeneous distribution of proteins revealed in situ in milk using confocal microscopy

An exciting new aspect of MFGM organization has emerged for the last 5 years, thanks to investigations performed in situ in milk using confocal laser scanning microscopy (CLSM; Evers et al. 2008; Lopez et al. 2010; Gallier et al. 2010; Lopez et al. 2011; Lopez and Ménard 2011; Zou et al. 2012, 2015; Nguyen et al. 2015). CLSM is a powerful tool to penetrate the surface of a sample and to visualize thin optical sections. It provides an opportunity to characterize milk fat globules in situ, without disturbing the internal structure, and to simultaneously localize the main elements of structure: TAG, phospholipids and membrane proteins. Using lipophilic fluorescent probes and lectins, Evers et al. (2008) reported heterogeneities in both the composition and structure of the MFGM within and among fat globules from the same species and also between milks from various mammal species. Using the exogenous fluorescent phospholipid Rh-DOPE, Lopez et al. (2010) observed both fluorescent and non-fluorescent areas at the surface of bovine milk fat globules and interpreted this heterogeneity as the phase separation of polar lipids in the outer bilayer of the MFGM (Fig. 1h–n). Lopez et al. (2010) hypothesized that this could correspond to the lateral segregation of MSM and cholesterol in rigid liquid-ordered (*Lo*) phase microdomains surrounded by the fluid matrix of glycerophospholipids in the liquid-disordered (*Ld*) phase. Milk-SM is 20 to 45% *w*/*w* of MFGM polar lipids depending on species (Lopez 2011). It contains long chain saturated fatty acids that are responsible for its high temperature of phase transition (Murthy et al. 2015) and is quantitatively the most saturated polar lipid in the MFGM. Moreover, sphingomyelin is known to associate with cholesterol in biological membranes to form domains called “lipid rafts” (Simons and Ikonen 1997). It is therefore hypothesized that the milk SM-rich microdomains revealed in the MFGM could originate from the lipid rafts of the apical plasma membrane of the lactating cells. The heterogeneous distribution of the Rh-DOPE dye in the MFGM has been confirmed by other research groups, *in situ* in bovine milk (Gallier et al. 2010; Zou et al. 2015) and in bovine colostrum (Zou et al. 2015). The lateral segregation of polar lipids in the MFGM was also characterized in human (Lopez and Ménard 2011; Zou et al. 2012) and in buffalo (Nguyen et al. 2015) milks. Differences in the size and pattern of the SM-rich domains have been characterized between fat globules from the same milk and in milks from various mammal species (Lopez et al. 2010; Lopez et al. 2011; Lopez and Ménard 2011) (Fig. 1j–n). A lateral diffusion of the SM-rich domains has been characterized in the plane of the MFGM as a function of time, assimilating them to moving platforms (Lopez et al. 2010; Nguyen et al. 2015). CLSM experiments also revealed that proteins and glycoproteins are heterogeneously distributed in the MFGM and are organized as patches or networks. Notably, the milk SM-rich microdomains are devoid of proteins (Evers et al. 2008; Lopez et al. 2010; Nguyen et al. 2015) (Fig. 1f–h).

On the basis of experimental results obtained by confocal microscopy, our group has proposed a new 3-dimensional and 2-dimensional model for the structure of the MFGM (Lopez et al. 2010; Lopez et al. 2011; Lopez 2011). For the first time, this model proposes the non-random organization of polar lipids, proteins and glycolipids in the plane of the MFGM. It describes the coexistence of at least two lipid phases in the MFGM: (i) the fluid matrix in *Ld* phase composed of the unsaturated glycerophospholipids (PE, PC, PI, PS), proteins, glycoproteins and glycolipids and (ii) the lateral segregation of MSM and cholesterol in *Lo* phase domains (Lopez 2011). The presence of SM-rich domains across the two leaflets of the MFGM bilayer or only on the outer leaflet of the MFGM remains to be elucidated. Recently, Zheng et al. (2014) proposed an updated model, integrating the repartition of the individual polar lipids in the bovine MFGM. The fundamental understanding of molecular arrangements in the MFGM is still in an embryonic stage.

#### Topography and nanomechanical properties of milk lipid membranes investigated using atomic force microscopy

**Fig. 2:**
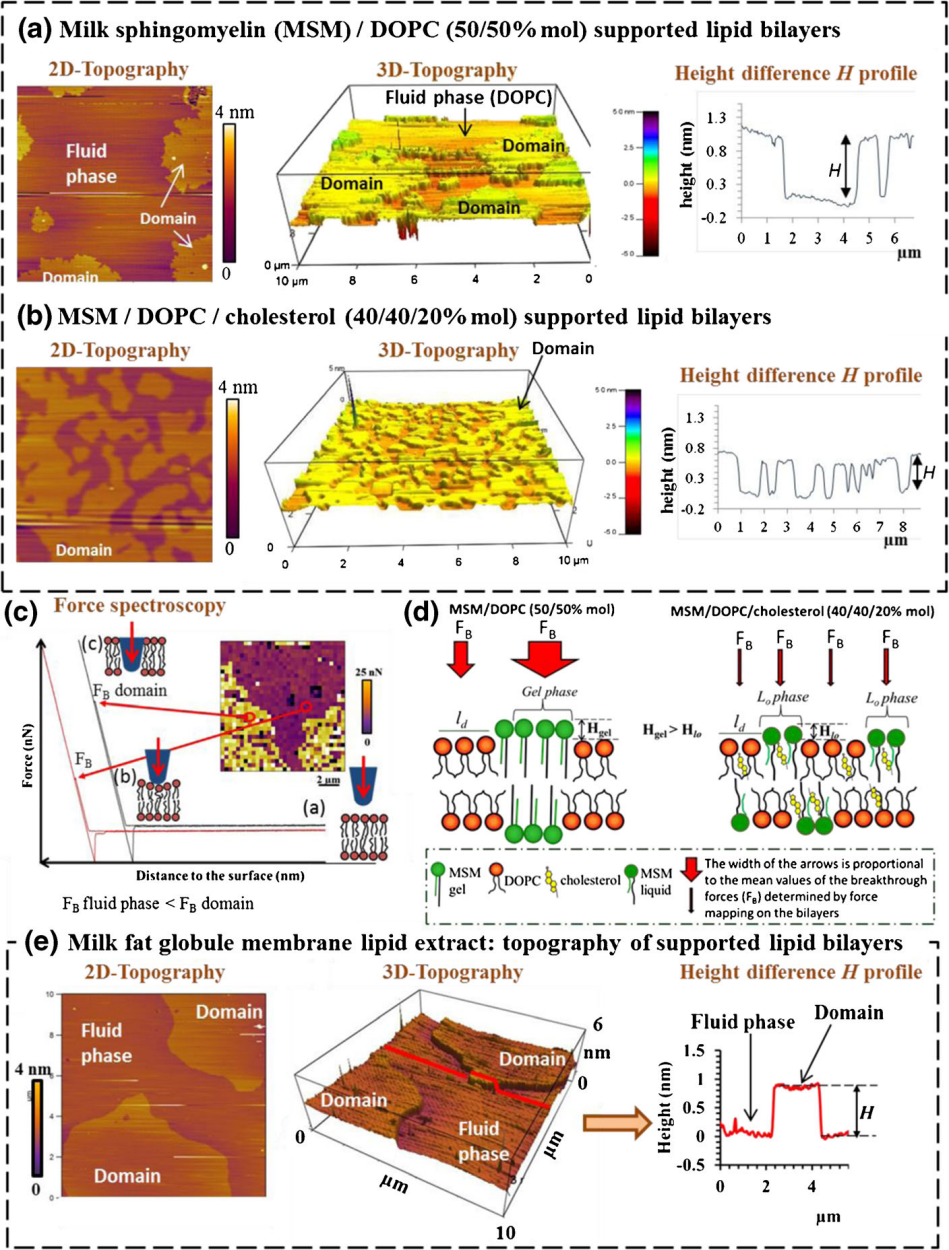
Topography and nanomechanical properties of supported lipid bilayers investigated by AFM imaging and force spectroscopy. AFM height images of supported lipid bilayers containing **a** MSM/DOPC, **b** MSM/DOPC/cholesterol, **e** MFGM lipid extract with 2-dimensional (*left*) and 3-dimensional (*middle*) representations. The colour scale bars to the right of the images provide height references for the images (*z*-range). *Right*: Cross-sectional line profiles showing height differences *H* between the domains and the fluid phase. **c** Typical force curves obtained on the different phases of a force map image of a lipid bilayer in fluid conditions (inserted image). **a** Away from the surface, the AFM tip is at rest and shows no applied force **b** when the AFM tip comes in contact with the surface, the applied force increases as the tip pushes against the membrane, **c** the membrane finally ruptures at a value of the force called the “breakthrough force” (F_B_) which differs from one phase to another depending on the local stiffness and stability of the membrane. **d** Molecular scheme of the supported lipid bilayers MSM/DOPC and MSM/DOPC/cholesterol with information on differences in the nanomechanical properties between domains (gel or liquid-ordered phases) and the fluid phase. Adapted from Guyomarc’h et al. (2014)

In the last decade, the emergence of atomic force microscopy (AFM) has opened large perspectives for the investigation of lipid membranes at the nanoscale. AFM imaging allows characterization of the topography of membranes with visualization of phase separations and measurements of the height difference between domains and the fluid phase. The idea of using AFM to investigate the surface nanostructure of interfacial films to better know the organization of the MFGM is recent and roots back into the first statement of interest by Evers (2004). The group of Rafael Jimenez-Flores (CalPoly, USA) first pioneered the deposition of MFGM components isolated from buttermilk powder to form a monolayer on a mica surface (Langmuir-Blodgett technique) and AFM imaging in air (Jimenez-Flores and Brisson 2008). The same group used this approach to show that polar lipids isolated from milk fractions or processed milk show domains at various surface pressures (Gallier et al. 2010, 2012). Recently, Murthy et al (2015) confirmed the formation of domains in MFGM lipid extract monolayers, protruding by about 1.5 nm at a surface pressure of 30 mN.m^-1^ and 20°C (i.e. below the phase transition temperature of MFGM polar lipids), and revealed the major role played by cholesterol on the topography of the membrane. However, this approach with monolayers may not account neither for the respective roles of the lipid components nor for the hydrated multilayer structure of the MFGM. For these reasons, the use of lipid mixtures with controlled composition (model systems or MFGM lipid extract) to form hydrated bilayers has been employed. Recently, our group has prepared supported lipid bilayers of milk sphingomyelin (MSM; main saturated polar lipid of the MFGM) and dioleoylphosphatidylcholine (DOPC; unsaturated polar lipid) and characterized the phase separation of these lipids for temperature T < 35°C with the formation of gel phase MSM domains protruding by 1 nm from the fluid DOPC phase (Fig. 2a; Guyomarc’h et al. 2014). The investigation of bilayers of MFGM lipid extract also allowed the visualization of domains protruding by ∼1 nm from the fluid phase (Fig. 2e). Therefore, the MSM was identified as a major component of the MFGM domains. With the ternary system MSM/DOPC/cholesterol (40:40:20% mol), the authors showed that the MSM-rich domains became dispersed (Fig. 2b; Guyomarc’h et al. 2014). Furthermore, AFM investigation into lipid bilayers allows the quantification of the nanomechanical properties of membranes (Fig. 2c; Garcia-Manyes and Sanz 2010; Li et al. 2011). Using this approach, the authors showed that the membrane’s resistance to rupture is significantly higher in the MSM domains as compared to the surrounding fluid DOPC phase (Fig. 2d; Guyomarc’h et al. 2014). Moreover, the authors showed that the addition of cholesterol to MSM/DOPC bilayers decreased the membrane’s resistance to rupture, probably through the conversion of the gel phase into liquid-ordered state (Fig. 2d; Guyomarc’h et al. 2015). Ongoing research is detailing the effect of cholesterol and temperature in bilayers of MFGM extract.

#### Updated model of the milk fat globule membrane

**Fig. 3:**
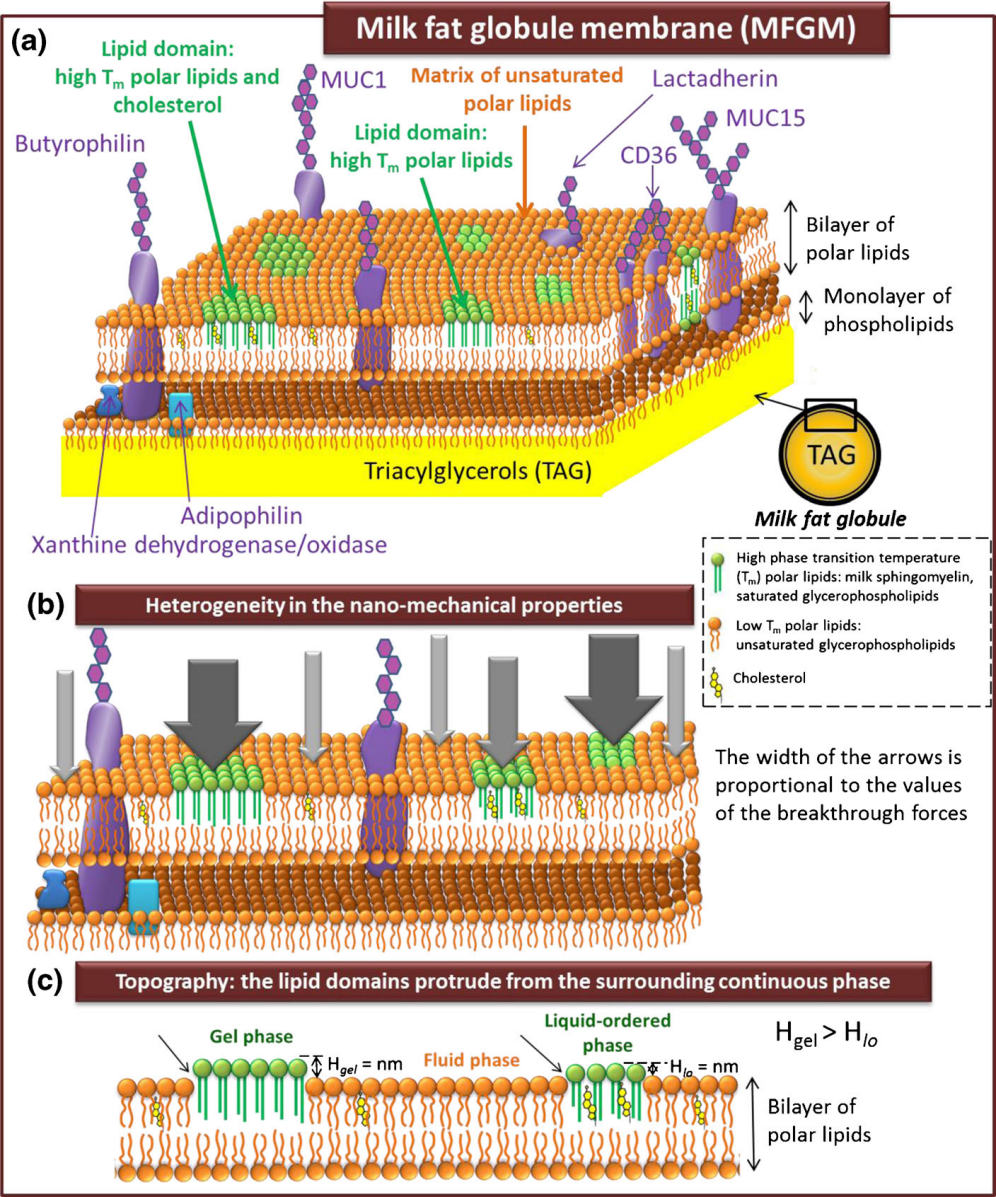
Updated model of the organization of the biological membrane surrounding fat globules in milk, the milk fat globule membrane (MFGM). **a** 3-Dimensional representation of the trilayered membrane, with the heterogeneous distribution of proteins and the phase coexistence of lipids: lateral segregation of high Tm polar lipids in domains. **b** Heterogeneities in the nano-mechanical properties of the outer bilayer of the MFGM. The width of the arrows is proportional to the mean values of the breakthrough forces determined by atomic force mapping. **c** Schematic representation showing that the MFGM is not flat. Hgel and Hlo are the average height difference between the two lipid phases

From the recent knowledge about the lateral segregation of polar lipids in the MFGM, we can propose an updated model of the organization of the MFGM (Fig. 3). Compared with the previous model (Lopez 2011), this model integrates (i) the fact that the domains can be formed by high phase transition temperature polar lipids (e.g. Milk-SM, DPPC) in the gel phase and/or by mixtures of high phase transition temperature polar lipids and cholesterol in the liquid-ordered phase, (ii) that the morphology (size, shape) of the domains and their height difference with the surrounding fluid phase depend on their lipid composition and mainly on the presence of cholesterol and (iii) that the nanomechanical properties are heterogeneous in the MFGM with larger resistance to rupture in the milk-SM rich domains (Fig. 3).

### Processed lipid droplets in milks and infant milk formulas

The fluid milks found in the supermarket for human consumption (both infants and adults) and the infant milk formulas (powders or liquid) are structurally far from the milk produced by female mammals. These structural changes could have processing implications, could affect the digestion and absorption of milk components and have nutritional and health consequences. This part of the paper shows the effect of technological processes, mainly homogenization, on the structure and interface composition of processed lipid droplets found in milks and infant milk formulas.

#### Processed lipid droplets in milks

**Fig. 4:**
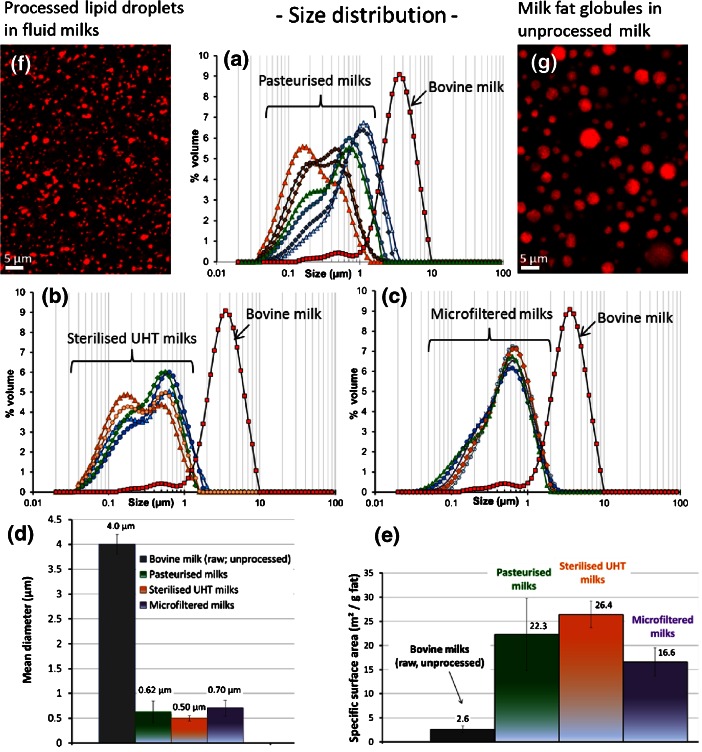
Microstructure of unprocessed bovine milk vs processed lipid droplets in commercial milks. Laser light scattering experiments showing the size distributions of lipid droplets **a** in pasteurized milks, **b** in sterilized UHT milks and **c** in microfiltrated milks, as compared to unprocessed bovine milk fat globules. Comparison of **d** the mean diameter and **e** the surface area between processed lipid droplets and milk fat globules in unprocessed milk. Confocal laser scattering microscopy images showing the size distribution of **f** processed lipid droplets in commercial processed milk and **g** milk fat globules in unprocessed milk (labeling of total fat by using Nile Red fluorescent dye; *red colour*)

Raw bovine milk is rarely consumed due to its short shelf-life and to fears regarding possible microbiological risks. Commercially available milks are therefore submitted to various technological processes before their consumption, e.g. thermal and mechanical treatments, standardization of fat content. Commercial milks are heat treated (pasteurized or heated at ultra-high temperature; UHT) or microfiltered (microorganisms are physically retained in the retentate by using 1.4 μm pore size membranes) for safe human consumption and to increase storage in the fridge or at room temperature. Fat standardization in commercial milks leads to the preparation of full-fat milks, semi-skimmed milks or skimmed milks. Also, commercial milks undergo a mechanical treatment, e.g. homogenization (pressures applied during industrial process: 5 to 20 MPa). The objective of homogenization is to reduce the size of milk fat globules in order to increase the physical stability of fat for long shelf-storage of the milks and to decrease the perception of excess fat intake due to the fat layer that develops on the milk surface when left to stand (fat globules rise to the top since fat is less dense than the aqueous phase of milk). Figure 4 shows the size distribution of lipid droplets in pasteurized, UHT and microfiltered milks as compared to the size distribution of fat globules in unprocessed bovine milk. The specific surface area of lipid droplets in unprocessed bovine milk is ∼2.6 ± 0.2 m^2^.g^-1^ fat while it is 16.6 ± 3.0 m^2^.g^-1^ fat in microfiltered milks (from 13.4 to 20.4 m^2^.g^-1^ fat; *n* = 7 milks), 22.3 ± 7.5 m^2^.g^-1^ fat in pasteurized milks (from 10.7 to 33.7 m^2^.g^-1^ fat; *n* = 32 milks) and 26.4 ± 2.7 m^2^.g^-1^ fat in UHT milks (from 23.2 to 34.9 m^2^.g^-1^; *n* = 21 milks). In conclusion, there are important differences in the size distribution of lipid droplets between commercially available processed milks and unprocessed bovine milk, with a significant lower size of lipid droplets in processed milks due to homogenization processing. Also, homogenization of milk leads to the disruption of the MFGM and to adsorption of milk proteins (caseins and whey proteins) at the TAG/water interface. After homogenization, the TAG/water interface consists of residual MFGM plus adsorbed milk proteins, of which caseins is the predominant group and whey proteins are mainly represented by β-lactoglobulin (see review Michalski and Januel 2006). Also, heat-denatured whey proteins can interact with MFGM proteins. The sequence of homogenization and heating changes the structure and the composition of the lipid/water interface. The surface increase of the TAG/water interface and changes in the interface composition are both important with respect to chemical reactions (e.g. susceptibility of products to Cu, light-induced flavour deterioration, changes in colour and flavour characteristics) and enzymatic reactions that occur at the interface. The impact on TAG lipolysis and thus the digestibility of fat globules are discussed in the paragraph 4 of this overview.

#### Processed lipid droplets in infant milk formulas

Breastfeeding is highly recommended for infants up to 6 months of age. When mothers cannot or do not want to breastfeed their infant or express their milk, infant milk formula may be used. Infant formulas are manufactured foods designed for the bottle-feeding of babies from powder or liquid. The composition of infant milk formulas is designed to be roughly based on a human mother’s milk composition at approximately 1 to 3 months *postpartum*. The most commonly used infant formulas are enriched in cow’s milk whey proteins and also contain casein as a protein source (or hydrolyzed proteins to facilitate digestion). They contain a blend of vegetable oils (e.g. palm oil, coconut oil, rapeseed oil and sunflower oil) or mixtures of vegetable oils with bovine milk fat as a fat source (the different sources of fats are combined to mimic the fatty acid composition of human milk), lactose as a carbohydrate source, vitamins, minerals and other ingredients depending on the manufacturer (e.g. prebiotics). Emulsifiers and stabilizers are commonly used to prevent the separation of the oil phase from water in the reconstituted infant milk formula. They include citric acid esters of monoglycerides and diglycerides, lecithins (from vegetable sources such as soya, from fish and krill), gums and maltodextrins.

**Fig. 5:**
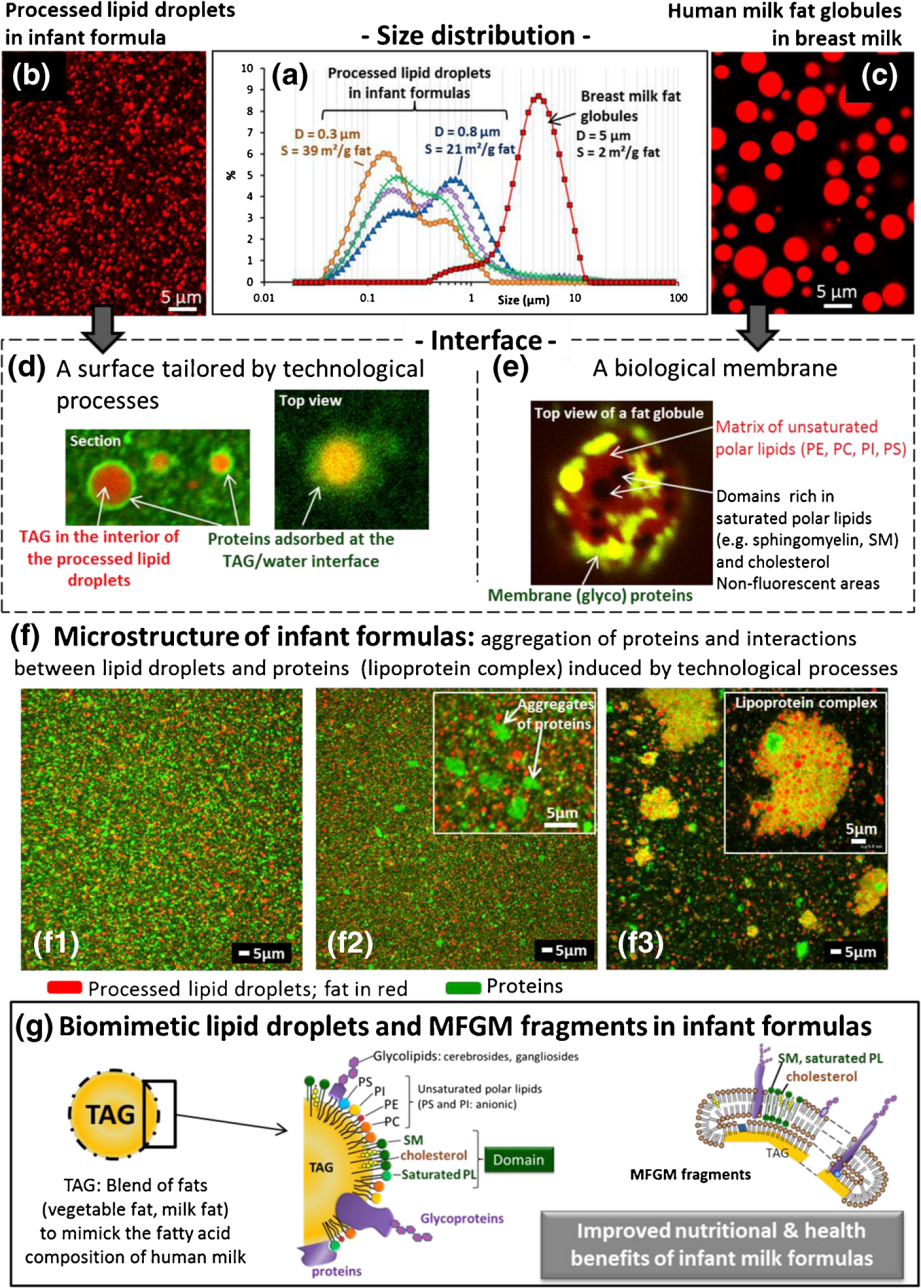
Comparison of the organization of lipids and interface architecture in human milk vs infant formulas. **a** Size distribution of milk fat globules in mature human milk vs processed lipid droplets in infant formulas, as determined by laser light scattering. Confocal laser scanning microscopy (CLSM) images showing the size distribution, composition and architecture of the interface of **b, d** processed lipid droplets in infant formulas and **c, d** milk fat globules in human milk. In images **b**, **c** and **d**, the labelling of total fat was performed by using Nile red fluorescent dye (*red colour*). In image **e**, the labelling of phospholipids in the biological membrane surrounding milk fat globules was performed by using Rhodamine-DOPE fluorescent dye. In images **d** and **e**, the proteins have been labelled by using Fast Green FCF fluorescent dye. **f** CLSM images showing the microstructure of infant formulas after hydration in a bottle. Total fat was labelled with Nile red fluorescent dye and proteins by Fast green FCF. **g** Schematic representation of biomimetic lipid droplets in infant formulas: presence of MFGM components (polar lipids, PL; membrane proteins) at the surface of processed lipid droplets

The microstructure of commercialized infant milk formulas has been investigated in this overview and compared to mature human milk (1 to 6 months *postpartum*). Figure 5 shows the differences in lipid droplet size and interface composition between human milk and infant milk formulas. The size distribution of mature breast milk spans from 0.4 to 13 μm with a mean diameter of 5 μm, corresponding to a surface of 2 m^2^.g^-1^ fat (Fig. 5a; Lopez and Ménard 2011). The processed lipid droplets found in the infant milk formulas have a significantly smaller size than breast milk fat globules (mean diameters: 0.3–0.8 μm; Fig. 5a–c) and a higher surface (from about 20 to 40 m^2^.g^-1^ fat; Fig. 5a). The small size of the lipid droplets found in infant milk formulas results from the homogenization step involved in the manufacture of these processed products. The objective of homogenization is to create an oil-in-water emulsion by mixing the blend of fats and the other components (proteins, minerals and emulsifiers). The pressure applied upon homogenization is adjusted to form small size droplets (i.e. mainly <1 μm) in order to ensure the physical stability of the processed emulsion during long storage of the powder and after hydration of the powder in the bottle. The differences in the size distributions of processed lipid droplets observed in different commercialized infant milk formulas (Fig. 5a) result from variation in the technological parameters used by different manufacturers or for different markets (i.e. differences in the homogenization process, concentration, evaporation and drying parameters or in the chemical composition). The composition of the TAG/water interface is also different between breast milk fat globules and the processed lipid droplets in infant milk formulas. The CLSM images presented in this overview clearly show the differences in interface composition: Processed lipid droplets in infant milk formulas are mainly covered by milk proteins (Fig. 5d) while breast milk fat globules are enveloped by polar lipids and membrane proteins organized in the MFGM (Fig. 5e), with occasional presence of cytoplasmic remnants (Fig. 1e, d; Lopez and Ménard 2011). Moreover, the structural analysis of various commercialized infant milk formulas revealed the presence of aggregates of proteins or of complexes formed between lipid droplets and proteins (Fig. 5f). These protein aggregates and lipoprotein complexes may result from the thermo-induced denaturation of proteins occurring during the heat treatments performed for the microbial safety of infant formulas. Such lipoprotein complexes induced by the industrial process raise questions about the accessibility of TAG and proteins by the digestive enzymes in the gastro-intestinal tract of newborns and then about the nutritional and health impacts.

The structural analysis presented in this paper clearly shows that the homogenization and the thermal treatments that are used to prepare the emulsion and for microbiological safety, respectively, have a great impact on the structure of fat and on the microstructure of the infant milk formulas. The structure of infant formulas is totally different from human milk, particularly the structure of fat (size of the lipid droplets, interfacial composition and architecture). The potential consequences on the mechanisms of digestion and absorption of lipids as well as on the metabolic programming are discussed in the paragraph 4.2 of this overview. The manufacture of biomimetic processed lipid droplets, with a size of the lipid droplets and a composition and architecture of their surface close to human milk fat globules, could improve the nutritional properties and health impact of infant formulas (Fig. 5g).

### Microstructure of dairy products showing the effect of technological processes on the organization of lipids

#### Cream

**Fig. 6:**
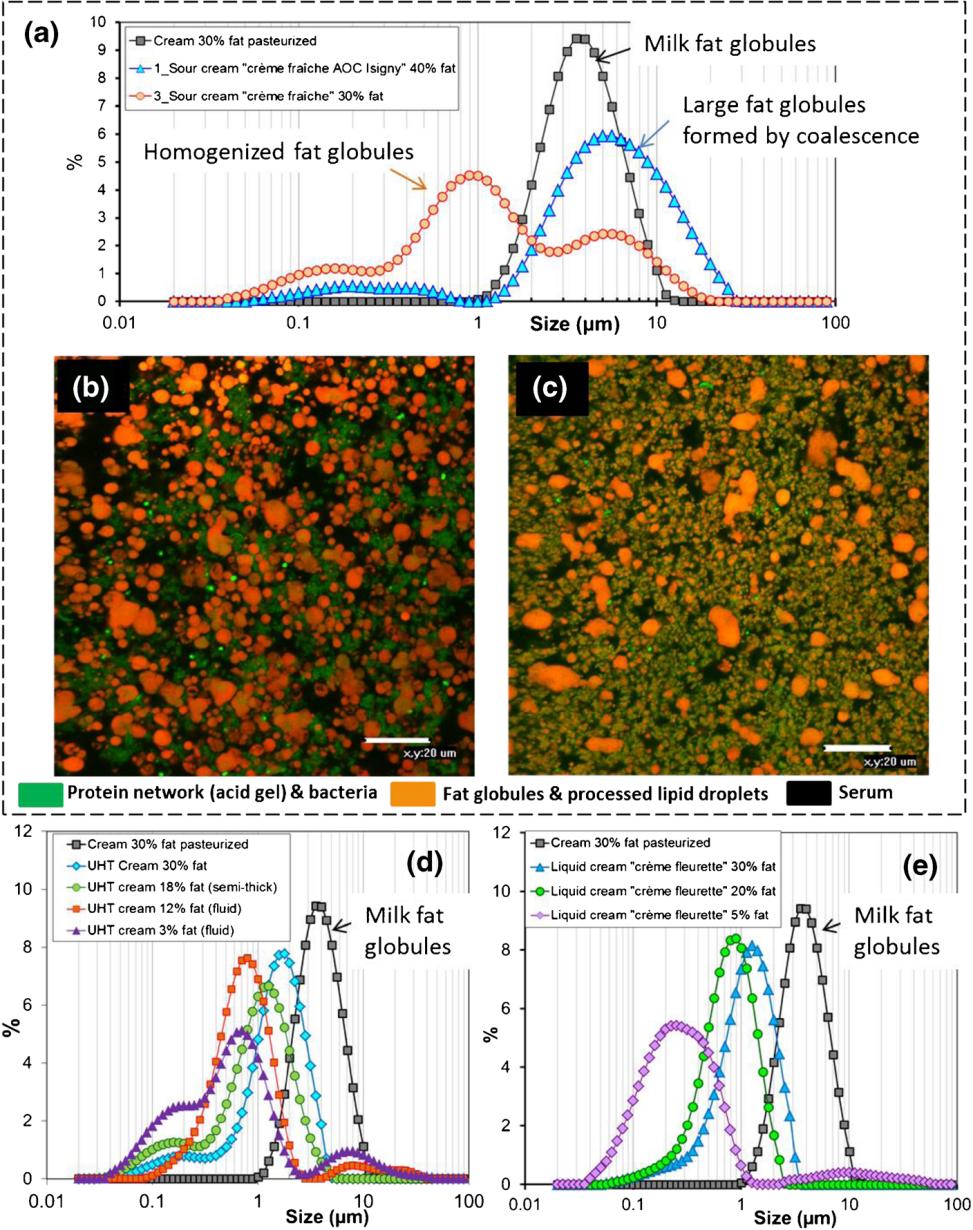
Size distribution of lipid droplets and microstructure of commercial dairy creams. **a** Size distribution of commercial soured creams vs unprocessed milk fat globules concentrated in a cream determined by laser light scattering. Confocal laser scanning microscopy images showing **b** the microstructure of a non-homogenized soured cream and **c** a homogenized soured cream. Total fat was labeled by using the Nile red fluorescent dye (*red colour*), proteins and bacteria were labeled by using acridine orange fluorescent dye (*green colour*). Size distribution of processed lipid droplets in **d** commercial UHT creams and **e** commercial liquid creams, revealing homogenization of the creams (e.g. processed lipid droplets with a size distribution lower than unprocessed milk fat globules concentrated in a cream)

Cream is a milk product, defined as an emulsion of milk fat-in-skim milk. In many countries, cream is sold in several grades, depending on the total fat content (heavy cream, light cream and extra-light cream), the viscosity (thickened and fluid) and the heat treatment applied (i.e. pasteurization and UHT) that governs the duration and temperature of storage of the cream. Commercial cream may contain added stabilizers and thickening agents (e.g. sodium alginate, carrageenan, xanthan gum, gelatin, corn starch, sodium bicarbonate, tetrasodium pyrophosphate and alginic acid). In the industrial production of dairy creams, the separation of fat globules from the aqueous phase of milk is accelerated using centrifuges called cream separators. Figure 6a shows the size distribution of milk fat globules in a cream obtained from milk by using a plate separator and pasteurized (fat content ∼300 g.kg^-1^). The size distribution of milk fat globules ranges from 1 to 12 μm with a mean volume-weighted diameter of 4.4 ± 0.2 μm (surface area = 1.8 m^2^.g^-1^ fat). Figure 6a presents the size distribution of lipid droplets in soured creams containing 30–40% *w*/*w* fat (thick fermented product; pH ∼ 4.5; “crème fraîche”). Figure 6b, c reveals the microstructure of industrial soured creams. Fat can be dispersed as native milk fat globules (traditional process), as shown Fig. 6b, or be at least partially homogenized, as revealed by the lower size of processed fat globules as compared to unprocessed fat globules (Fig. 6c). Microstructural analysis also shows that small processed lipid droplets covered by proteins are connected with the protein network formed by caseins at acidic pH. Bacteria used for culture can be observed in the product, as well as the protein network formed by proteins at pH 4.5 (Fig. 6b). Figure 6d, e shows the size distribution of processed lipid droplets in sterilized UHT creams and liquid pasteurized cream (crème fleurette), respectively. As compared to regular cream, the small size of the processed lipid droplets reveals that milk fat globules have been homogenized. Homogenization of cream is used to increase the physical stability of the cream upon storage (avoid the formation of a cream layer at the top of the product), to limit exudation of whey and to increase the viscosity of the product (and then avoid addition of thickeners). The pressure applied during homogenization, in general from 5 to 25 MPa, depends on the functional properties of the cream. Hence, most of the commercially available creams contain processed lipid droplets to reach functional properties.

#### Microstructure of butter, buttermilk and butter serum

Several technological steps are involved in the manufacture of butter: concentration of fat globules from milk (fat content from 40 g.kg^-1^ in milk to at least 400 g.kg^-1^ in the cream), pasteurization of cream, storage of cream at low temperature to induce TAG crystallization, acidification, churning of cream leading to the disruption of fat globules with phase inversion from oil-in-water to water-in-oil emulsion, production of small butter grains, pressing of the butter grains and then conditioning of butter (820 g.kg^-1^ fat).

**Fig. 7:**
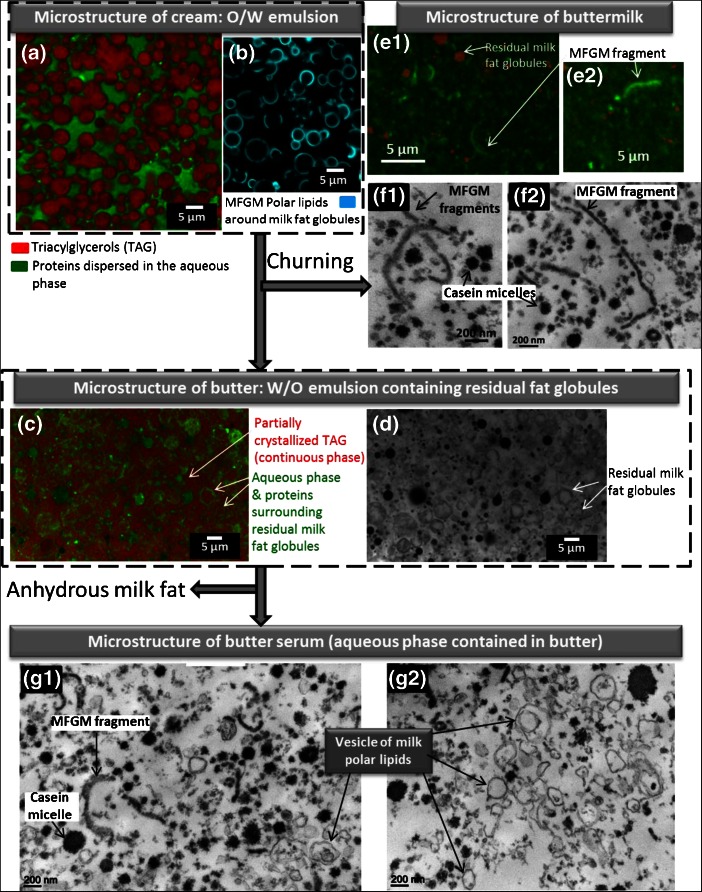
Changes in the organization of lipids upon the manufacture of butter. **a, b** confocal laser scanning electron microscopy (CLSM) images showing the microstructure of cream. **c, d** CLSM images showing the microstructure of butter. Microstructure of buttermilk characterized by using CLSM (**e**) and transmission electron microscopy (**f**). Microstructure of butter serum revealed by using TEM (**g**)

Figure 7 shows changes in the structural organization of lipids upon the manufacture of butter. The microstructure of a cream before churning, characterized by CLSM, shows concentrated milk fat globules enveloped by their biological membrane that are dispersed in the aqueous phase, e.g. an oil-in-water emulsion (Fig. 7a, b). The microstructure of butter, characterized by CLSM, shows pockets of serum and residual milk fat globules that are dispersed in the continuous phase formed by partially crystallized TAG (Fig. 7c, d).

Buttermilk and butter serum are the liquid phases released during churning of cream in the process of butter making and during preparation of anhydrous milk fat by melting of butter and then centrifugation to separate anhydrous TAG to the aqueous phase contained in butter, respectively. Buttermilk and butter serum contain about 2 and 11% *w*/*w* of polar lipids on dry matter, with 19 and 30% *w*/*w* of sphingolipids on polar lipids, respectively (Rombaut et al. 2006). Figure 7e, f shows the microstructure of buttermilk characterized using CLSM (Fig. 7e) and TEM (Fig. 7f). CLSM images show residual milk fat globules (a core of TAG covered by the MFGM) and butter grains as well as elongated structures that correspond to MFGM fragments (Fig. 7e; Fauquant et al. 2014). The resolution of TEM allowed observation of casein micelles and fragments of the MFGM released from the surface of milk fat globules by the mechanical process applied during churning of the cream. The size of the MFGM fragments released in the buttermilks, ranging from 1 to several micrometers, could be related to the size of the initial fat globule before churning. These TEM images are consistent with previous studies (Corredig and Dalgleish 1997; Morin et al. 2007). Figure 7g shows the microstructure of industrial butter serum composed by casein micelles (black particles with a circular shape in the images), MFGM fragments (elongated black particles) and vesicles of polar lipids (100–400 nm; circular shape with the polar lipid bilayer appearing in black; the interior of the lipid vesicles appears in white colour). These vesicles result from the alteration of the MFGM and self-organization of polar lipids in an aqueous environment. Differences in the microstructure of buttermilk and butter serum and differences in the organization of polar lipids (MFGM fragments vs vesicles of polar lipids, respectively) result from processing (mechanical treatment and temperature).

From this microstructural analysis and from chemical determinations, we learn that the different technology steps involved in the manufacture of butter and anhydrous milk fat lead to the fractionation of milk lipid components according to their polarity. Neutral lipids such as TAG are mainly recovered in butter while the polar lipids organized as vesicles and MFGM fragments are selectively found in the aqueous phases such as buttermilk and butter serum. Among polar lipids, the relative percentage of milk sphingomyelin is higher in butter serum than in buttermilk (Rombaut et al. 2006), which could be related to its specific biophysical properties (i.e. changes in its physical state as a function of temperature, Murthy et al. 2015).

Milk polar lipids have been reported to have interesting functional properties, such as emulsifying and foaming properties, and potentialities to form liposomes for encapsulation (Singh 2006; Dewettinck et al. 2008; Vanderghem et al. 2010). Also, polar lipids have been reported to have interesting nutritional properties and to provide health benefits (Dewettinck et al. 2008). Hence, in the last years, academic and industrials teams have focused on the development of technological processes aiming at purifying and concentrating MFGM fragments and milk polar lipids from buttermilk and butter serum to prepare milk polar lipid or MFGM-enriched ingredients (Dewettinck et al. 2008; Vanderghem et al. 2010; Gassi et al. 2016).

#### Microstructure of cheeses revealing the major role of processing on the organization of fat

Cheeses are complex dairy products composed of a matrix of protein entrapping fat, aqueous phase containing minerals and bacteria. The structure, texture and mouth feel of cheeses are highly dependent on the fat content within the cheese and the organization of fat. Moreover, the functional properties of cheeses and cheese ingredients (e.g. pizza-style cheeses) are dictated by structure. Hence, analyzing cheese microstructure is of primary importance (Everett and Auty 2008; El-Bakry and Sheehan 2014). Recent research has focused on the organization of fat and the role of the MFGM on cheese structure, texture, functional properties (stretching, melting and free oil formation) and flavour development (Lopez et al. 2006; Lopez et al. 2007; Everett and Auty 2008; Ong et al. 2011; Romeih et al. 2012; Hickey et al. 2015). CLSM associated with pertinent fluorescent probes has permitted (i) to gain information about the spatial organization of individual cheese components in situ in cheeses (TAG, polar lipids, protein network and bacterial colonies) and (ii) the identification of the key steps of the process that can drastically change the properties of fat (Lopez et al. 2006, 2007, 2008; Ong et al. 2011; Ma et al. 2013; El-Bakry and Sheehan 2014). Microscopy techniques showed that the organization of fat in cheeses results from the different steps involved in the technological process, from coagulation to ripening. Examples of different organizations of fat in cheeses, resulting from processing, are presented below.

**Fig. 8:**
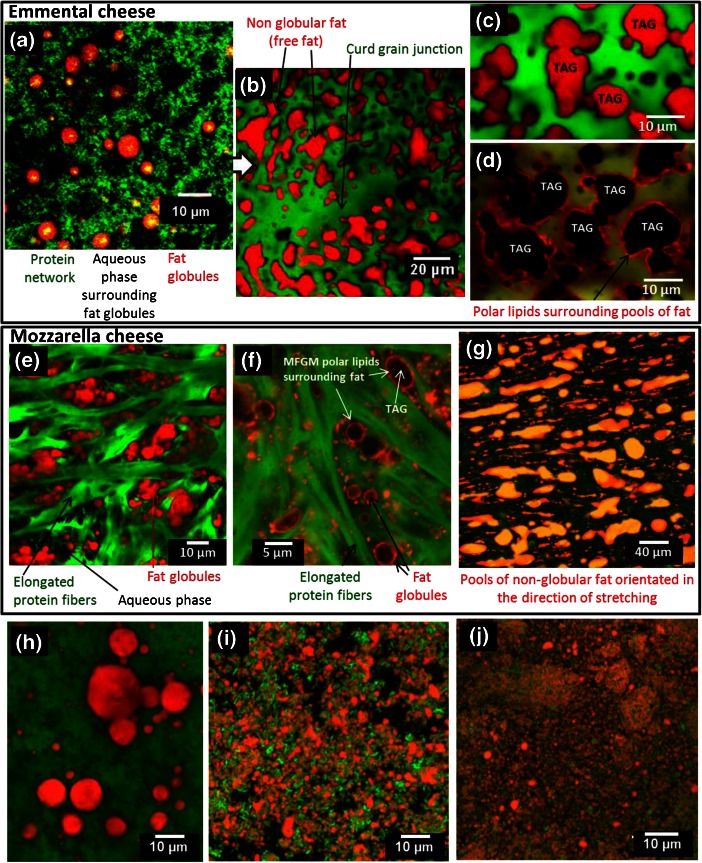
Microstructure of cheeses showing the effect of dairy technological processes on the organization of lipids. Confocal laser scanning microscopy (CLSM) images of (**a–d**) Emmental cheeses: **a** after coagulation, **b** after pressing of the curd grains, **c**, **d** in the final product, **e, f** high-moisture Mozzarella cheese, **g** low-moisture Mozzarella cheese, **h** mould-ripened soft-type cheese, **i** homogenized cream cheese, **j** homogenized fresh cheese. In all the images, proteins were labelled by using fast green FCF fluorescent dye (*green colour*). Images **a**–**c**, **e**, **g**, **h**–**j**: Total fat was labelled using Nile red (*red colour*). Images **d** and **f**: Phospholipids from the milk fat globule membrane were labelled using Rhodamine-DOPE fluorescent dye (*red colour*). Scales bars are in the images

Regarding hard-type cheeses, very interesting studies on the microstructure of Cheddar cheese have been conducted (Ong et al. 2012), showing destabilization of fat globules within the casein matrix. In Swiss-type cheeses (e.g. Emmental), fat globules are also altered during manufacturing (Fig. 8a–d). Coagulation of milk leads to the formation of a continuous network of casein strands in which fat globules are entrapped (Fig. 8a). Pressing of curd grains and high cooking temperatures up to 55°C results in the disruption of fat globules, coalescence and formation of non-globular free fat (Fig. 8b, c; Lopez et al. 2007). As revealed by CLSM with the fluorescent probe Rhodamine-DOPE, free fat within the Emmental cheese matrix is still covered by polar lipids originating from the MFGM (Fig. 8d; Lopez et al. 2008). The size of the free fat pools formed within the cheese matrix and the oiling-off can be modulated by technological parameters as the heat load applied during pressing and acidification (Richoux et al. 2008). The various structures of fat observed during Swiss-type cheese production and the organization of surface-active material at the interface have been reported (Lopez et al. 2006; 2008) and recently highlighted (Hickey et al. 2015). Studies showed that in hard-type cheeses the bacterial colonies are mainly located at the protein–fat interface, favoring generation of volatile flavour compound from the fat substrate, which influences the final flavour of the ripened cheese (Lopez et al. 2006 ; El-Bakry and Sheehan 2014).

In Mozzarella cheese, fat is located in the aqueous phase entrapped between the elongated protein fibers formed during the pasta filata process (stretching of the curd; Fig. 8e–g). The chemical composition of the cheeses and technological parameters alter the organization of fat (Ma et al. 2013). In high-moisture Mozzarella cheese (e.g. balls found in supermarkets), the structural analysis performed using CLSM showed that fat is organized as globules of about 4 μm in diameter that are mostly aggregated and partially coalesced (Fig. 8e). CLSM performed with the fluorescent probe Rhodamine-DOPE revealed that fat globules are mainly covered by their biological membrane (i.e. the MFGM) (Fig. 8f). In industrial low-moisture Mozzarella cheeses (i.e. pizza cheese ingredient), fat is mainly organized as pools of non-globular fat orientated in the direction of stretching (Fig. 8g), as already reported (Everett and Auty 2008; Ma et al. 2013). These pools of fat result from the disruption of fat globules and their subsequent coalescence due to high temperature cooking, mechanical stretching conditions during manufacturing and ripening. Fat globules covered by the MFGM and pools of fat still covered by polar lipids do not interact with the casein network in the Mozzarella cheese matrix. The size of the fat globules in Mozzarella cheese was positively correlated to meltability and free fat formation (Ma et al. 2013).

The technological steps involved in the manufacture of mould-ripened soft cheeses do not alter greatly the organization of fat. For example, Fig. 8h shows the microstructure of a mould-ripened soft cheese in which milk fat globules are individualized in the casein network (diameter from 4 to 10 μm) or aggregated. Aggregation of milk fat globules facilitates their coalescence and then the increase in the size of milk fat globules (Fig. 8h).

Homogenization of milk is involved in the manufacture of some cheeses, e.g. some fresh cheese or cream cheese. Figure 8i, j shows the microstructure of two commercialized cream cheeses, in which fat globules have been homogenized. The mean diameter of processed lipid droplets is around 1 μm. CLSM images clearly show the interactions between the proteins adsorbed at the lipid/water interface and the proteins in the surrounding phase.

**Fig. 9:**
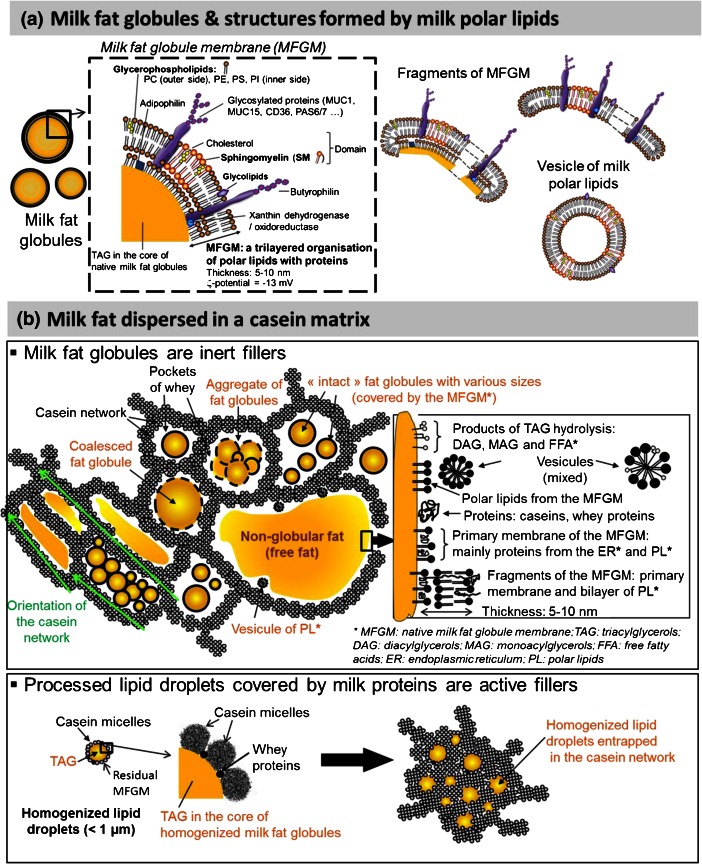
Microstructure of milk fat components. **a**, *left*: Milk fat globules surrounded by their biological membrane, the milk fat globule membrane (MFGM): a trilayer of polar lipids and proteins. **a**, *right*: Structures derived from the MFGM: fragments of MFGM and vesicles of milk polar lipids characterized in buttermilks and butter serum, respectively. **b** Schematic representation of the organization of milk fat in a casein matrix, as observed in various dairy products such as acidified dairy creams, yoghurt and cheeses

As a conclusion, various organizations of lipids can be present within the cheese matrix, depending mainly on the mechanical treatments (homogenization and/or draining operations) applied during the manufacture of cheeses (Fig. 9). Fat can be dispersed as (i) fat globules of 4 μm in diameter covered by their biological membrane (e.g. high-moisture Mozzarella cheese, mould-ripened soft cheeses, i.e. no homogenization, moderate drainage), (ii) inclusions of non-globular free fat of several μm (e.g. Cheddar cheese, Emmental cheese, low-moisture Mozzarella cheese, i.e. no homogenization and intense drainage), (iii) very small homogenized lipid droplets covered by milk proteins (e.g. fresh cheeses, cream cheese, blue cheeses).

## The processing of milk affects the microstructure of fat and alters the biological membrane: discussion on the potential impacts

### Technological processes alter milk fat globules and their role in structure and texture: inert vs active fillers

This overview showed that processing of milk alters milk fat globules, changes their size and the architecture of their surface (composition and organization). Also, churning of milk fat globules releases MFGM fragments that can form milk polar lipid vesicles (Fig. 9). The composition of the TAG/water interface (e.g. MFGM, monolayer of phospholipids, caseins and whey proteins) governs the interactions of milk fat with the surrounding protein matrix, leading to specific structural and rheological properties (concept of inert fillers or active fillers; Fig. 9). Changes in the size of the lipid droplets and of the TAG/water interface composition also affect the mechanisms of milk fat crystallization, but this is out of the scope of this overview.

In all cheese matrices where MFGM remnants, polar lipids and/or lipolysis-induced mono- and diglycerides occupy the TAG/water interface, no interaction is seen between the milk fat and the protein network (Fig. 9). Fat is then qualified an “inert filler” in the casein matrix (i.e. have little or no ability to interact; Fig. 9). This is the case for all processes that do not involve homogenization (e.g. hard-type cheese, mozzarella cheese and mould-ripened cheese; Fig. 8).

**Fig. 10:**
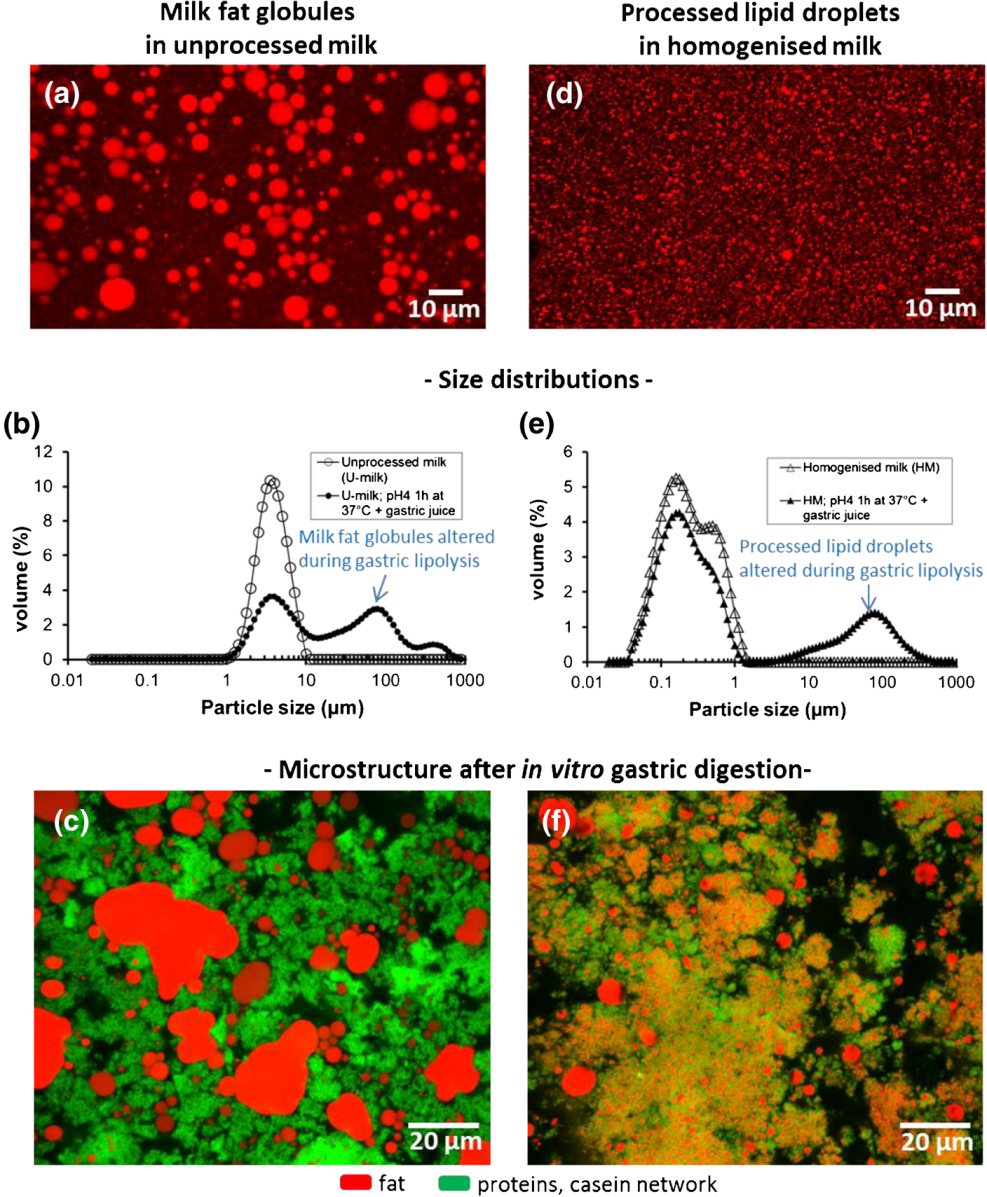
Structural evolution of milk fat globules in unprocessed milk vs processed lipid droplets in homogenized milk upon simulated human gastric digestion. Confocal laser scanning microscopy (CLSM) images showing the size distribution of the lipids droplets **a** in unprocessed milk, **d** in homogenized milk. Particle size distribution measured in the initial milks and after gastric digestion for **b** unprocessed milk and **e** homogenized milk. CLSM images showing the microstructure upon gastric digestion of **c** unprocessed milk and **f** homogenized milk. Adapted from Garcia et al. (2014)

The structural analysis of various milks and dairy products showed that homogenization is often used in the dairy industry (commercialized milks, Fig. 4; infant formulas, Fig. 5; creams, Fig. 7; cream cheeses, Fig. 8i, i). The main objective of homogenization is (i) to improve the physical stability of the emulsion (example of commercially available milks), (ii) to increase the viscosity of the product, i.e. improve the texture (example of dairy creams), (iii) to create an emulsion (example of the infant formulas) and (iv) to promote lipolysis (blue cheeses). The pressures ranging from 5 to 25 MPa at the industrial scale lead to the formation of small (<1 μm) processed lipid droplets, to the disruption of the MFGM and to the adsorption of milk proteins (mainly caseins) to cover the newly formed interface (Fig. 9). After homogenization, the caseins adsorbed at the TAG/water interface interact with the casein matrix formed (i) by renneting during cheese-making (Fig. 9i, j) or (ii) at acidic pH for example in the sour dairy creams (Fig. 7c), but also in the stomach upon digestion of milk and dairy products (Fig. 10). Homogenized processed lipid droplets covered by caseins are considered as active fillers (Fig. 9), providing a structural role and increasing firmness of the protein matrix (Everett and Auty 2008). Hence, the composition of the fat globule surface affects (i) the interactions between lipid droplets and the protein network, (ii) the structure of the matrix and (iii) the rheological properties and texture of the product.

For the next decade, the science of the emulsions will bring new opportunities to the dairy industry. The preparation of emulsions with various sizes and specific components located at the TAG/water interface will permit modulation of the interactions with the protein network (inert vs active fillers). Also, the tailoring of emulsions covered by MFGM-originating components (milk polar lipids, cholesterol and MFGM-proteins) should be further considered to prepare small lipid droplets, mimicking the interfacial properties of unprocessed milk fat globules.

### Secreted milk fat globules vs processed lipid droplets in milks and infant milk formulas: industry may learn from biological lipid assemblies to improve the nutritional and health impacts

Milk is a complex arrangement of multiple components that are digested and absorbed though a dynamic and interrelated process, rather than as individual components, highlighting the importance of food microstructure in delivering health benefits.

Milk processing, mainly homogenization, affects the structure of fat as compared to the unprocessed fat globules found in milk: The size decreases, the surface area increases and the architecture of the TAG/water interface changes (bovine or human milk fat globules vs processed lipid droplets; Figs. 4 and 5). Studies showed the importance of the properties of the TAG/water interface, mainly the composition and amount of interface (expressed in m^2^ per g of fat; inversely related to the diameter of lipid droplets), in the adsorption and activity of digestive enzymes. It is now well accepted that the native milk fat globules and the processed lipid droplets will evolve differently in the gastrointestinal tract, with consequences in digestive lipolysis and potential impacts on lipid bioaccessibility, absorption and on the postprandial fate of dietary fatty acids.

#### The accessibility of lipids by the digestive enzymes governs their hydrolysis and metabolic fate: importance of the TAG/water interface amount and architecture and role played by the microstructure upon gastric digestion

Recent studies focused on the digestibility of lipids in milk as a function of their organization, which results from dairy processes (mainly homogenization and heat treatments). Homogenization of milk affects both the size of lipid droplets and the architecture of the TAG/water interface (milk fat globules: 4 μm in diameter, covered by the MFGM vs processed lipid droplets: <1 μm diameter, covered by proteins). It is then difficult to dissociate the effects due to the size of the lipid droplets from those due to the composition of the interface. Garcia et al. (2014) investigated the effect of the droplet size (native milk fat globules of various sizes, homogenized milk lipid droplets) and interface composition (MFGM vs adsorbed milk proteins), in vitro, in conditions close to human physiology. The authors showed that the digestion of TAG is twice as efficient for small native fat globules (1.75 μm; 4.3 m^2^.g^-1^ fat) as for large native milk fat globules (6.6 μm; 1.6 m^2^.g^-1^ fat) in the gastric phase and is about 30% higher in the intestinal phase. This study provided evidence that, for a similar interface composition (i.e. the MFGM), the amount of the interface directly influences the digestion of milk fat globules (see Lopez et al. 2011 for details about the preparation and composition of size-partitioned milk fat globules). With the same milk (i.e. exactly the same chemical composition), the effect of homogenization on the in vitro digestion of milk lipids has been investigated (Berton et al. 2012; Garcia et al. 2014). The changes in the interface composition (i.e. adsorption of casein micelles and whey proteins) upon decreasing globule size by homogenization had opposite effects to those found when decreasing the diameter of milk fat globules without altering the MFGM, leading to lower levels of lipolysis as expected (Garcia et al. 2014). Heat treatment (UHT sterilization) did not further affect the digestibility of homogenized milk. Structural characterizations performed during in vitro digestion revealed (i) an increase in the size of both native milk fat globules and homogenized lipid droplets upon gastric lipolysis and (ii) differences in the microstructure after coagulation of milk proteins at acidic pH (Fig. 10; adapted from Garcia et al. 2014). It is possible that the interactions between the caseins adsorbed at the surface of homogenized lipid droplets and the protein network have formed upon coagulation of milk in the stomach (Fig. 10f). This would decrease the accessibility for the gastric lipase to TAG and then decrease the levels of lipolysis. Then, the concept of processed lipid droplets as active fillers in the stomach could be responsible for lower levels of lipolysis as compared to inert fillers (i.e. unprocessed milk fat globules). The physical stability of the emulsion in the stomach has also been reported to affect the gastric lipolysis rates and gastric emptying (Golding et al. 2011). Investigations of the effects of milk lipid droplet size and interface composition (MFGM vs. milk proteins) on the activity of the human pancreatic lipase revealed a higher lipolysis for small processed lipid droplets but a lower catalytic efficiency than for milk fat globules covered by the MFGM (twice as much lipolysis despite the 25-fold larger available surface in homogenized milk; Berton et al. 2012). Studies performed in the rat showed that processing of milk (homogenization and heat treatment) affect the microstructure of fat upon digestion (Gallier et al. 2013a, b).

In conclusion, the main parameters affecting the digestibility of milk lipids are the size of the lipid droplets, the composition of the TAG/water interface and the microstructure of the stomach bolus at acidic pH. The role played by the microstructure of milk in the stomach (liquid *vs* coagulated; protein–lipid droplets interactions) could be a means to modulate digestion of lipids.

#### Processed lipid droplets in infant milk formulas vs breast milk fat globules: when the structure and architecture of the interface really matters for infants

Breast milk or infant milk formulas are the exclusive foods for infants from birth to about 6 months of age. The milk lipids supply 50 to 60% of the calories necessary for newborn growth. Hence, the efficiency of milk lipid digestion and absorption is of particular importance for infants. The differences in TAG composition and internal structure (position of fatty acids on the three glycerol carbons) between human milk and infant milk formulas could affect the digestibility of infant formulas, but it is out of the scope of this overview focused on the organization of lipids at a microscopic level. The pioneering study of Armand et al. (1996) showed in premature newborns that the digestibility of TAG in human milk (native milk fat globules; 4 μm) is more efficient than in infant milk formulas (processed lipid droplets; 0.6 μm). Namely, 25% of TAG in breast milk was hydrolyzed in the gastric phase after 50 min vs 14% for formulas. The absorption of lipids was also higher with human milk as compared to the infant milk formula (Armand et al. 1996). Through this in vivo study, the group of Martine Armand showed that the size of lipid droplets is not the main parameter involved in the digestion of TAG. The unique composition and structure of the biological membrane surrounding fat globules in milk could play a specific role in the mechanisms of adsorption and hydrolysis of TAG by the digestive enzymes, as well as on the metabolic fate of milk lipids. The lateral packing of polar lipids in the outer bilayer of the MFGM (i.e. phase coexistence of SM-rich domains in the gel or liquid-ordered phase in presence of cholesterol and a surrounding fluid phase; Fig. 1) could be involved in the mechanisms of milk fat globule digestion (i.e. adsorption and activity of the digestive enzymes; gastric lipase, bile salt stimulated lipase, pancreatic lipase), as previously discussed (Lopez et al. 2010; Lopez 2011; Lopez and Ménard 2011). Also, the composition of the TAG/water interface can affect the microstructure of milk and infant milk formulas after coagulation of caseins occurring at acidic pH in the stomach (same mechanism as Fig. 10). Breast milk fat globules are inert fillers while processed lipid droplets covered by milk proteins in infant milk formulas are active fillers, which can affect the accessibility of lipids for their digestion and absorption with nutritional impact in infants.

The presence of the MFGM around fat globules or its absence in infant milk formulas is an outstanding question. The microstructure of lipids in infant milk formulas could be closer to breast milk by increasing the size of the processed droplets and by adding MFGM components at the TAG/water interface (Fig. 5g). Infant milk formula manufacturers are developing such new concept of infant formulas with a structural organization of lipids close to breast milk. Oosting et al. (2012) showed that feeding young mice with large lipid droplets coated by milk phospholipids reduced fat accumulation and improved metabolic profile in adulthood as compared to a control infant formula. This study provides a proof of concept that the size and the presence of MFGM components at the surface of processed droplets of early dietary lipids contribute to body composition and metabolic health later in life, then on metabolic programming (Oosting et al. 2012). Moreover, recent studies revealed a direct effect of the presence of MFGM in infant formulas on the cognitive development of infants (Timby et al. 2014). Milk fat globules could supply to the infant the necessary instructions for the development of the intestinal mucosa, of the immune and nervous systems as well as for metabolic activity (Riccio 2004). Moreover, some components of the MFGM are involved with anti-infection or anti-adhesion properties and, hence, with protection of the newborn from various viral and bacterial infections (see reviews: Ward et al. 2006; Dewettinck et al. 2008; Lopez 2011). Therefore, we should consider that both the chemical composition and the structure of biological entities such as milk fat globules components may have evolved over time and may have been optimized through the selective pressure that occurred specifically for each mammal species to assume essential biological functions.

For the next decade, the technological challenge will be to manufacture infant milk formulas containing processed lipid droplets with structural characteristic, interfacial properties and functions close to those of native milk fat globules, i.e. biomimetic lipid droplets (Fig. 5g). To reach this objective, MFGM components concentrated and purified from buttermilk or butter serum (by-products of the bovine dairy industry) could be used. The formulation of milk polar lipids in replacement of non-dairy emulsifiers (e.g. soya lecithin) will bring sphingolipids (milk sphingomyelin, cerebrosides and gangliosides) and bioactive membrane proteins for a positive impact in the gastrointestinal tract of infants. Moreover, the active role played by cholesterol on the physical properties of milk sphingomyelin should be considered in the formulation of biomimetic infant milk formulas (Guyomarc’h et al. 2014; Murthy et al. 2015). These biomimetic lipid droplets will certainly improve the nutritional and health benefits of infant milk formulas.

### A specific role played by the biological membrane enveloping milk fat globules on the nutritional properties and health impact of milk fat?

Milk fat has long been recognized as having cholesterol-raising effects that are caused by the high content of long-chain saturated fatty acids, especially when compared with that of vegetable oils (Iggman et al. 2011). However, epidemiological studies suggested that the association between milk fat intake and cardiovascular disease is partly dependent on the type of dairy food consumed (Goldbohm et al. 2011; Patterson et al. 2013). Controlled studies showed that butter causes higher cholesterol concentrations than do cheeses, even with a similar intake of milk fat (Hjerpsted et al. 2011). The composition (fat, protein and calcium contents), viscosity and microstructure (liquid vs. gel vs. butter) of the food matrix as well as the organization of fat (bulk, emulsified and size of lipid droplets) and its physical state (liquid *vs* crystallized) may possibly modulate the mechanisms of digestion and hypercholesterolemic effects of saturated fat (Michalski et al. 2013). Moreover, recent studies hypothesized that the different effects of various dairy foods on plasma lipids might be caused by the presence of the MFGM, which is rich in bioactive membrane proteins generally considered to exhibit favourable metabolic effects (Spitsberg 2005) and polar lipids (e.g. sphingolipids, mainly MSM). Animal studies showed that MFGM and sphingolipids could lower plasma cholesterol partly by modulating hepatic gene expression (Kamili et al. 2010; Zhou et al. 2012; Chung et al. 2013).

This overview shows that the MFGM is preserved differently in dairy products as a consequence of technological processes that can alter milk fat globules and then alter the physical structure of this biological membrane (Figs. 4–8). The question is: Are the potential beneficial effects due to the whole MFGM surrounding fat globules (i.e. with a trilayered organisation and specific biophysical properties) or to individual components of the MFGM that can be found in MFGM-enriched ingredients? Conflicting results are found in the literature. A recent study showed no effect of MFGM-enriched ingredient on postprandial lipid concentrations (Ohlsson et al. 2010) whereas a study using buttermilk showed significant reductions in both fasting total cholesterol and LDL-cholesterol (Conway et al. 2013). A very recent study compared native milk fat globules covered by their physically intact MFGM with butter oil to determine the effects on lipid profiles (Rosqvist et al. 2015). The results show that the presence of MFGM may counteract the hypercholesterolemic effects of saturated fat. The mechanisms behind the potential effects of the MFGM on plasma lipids are unclear but may involve reduced cholesterol absorption or phospholipid-induced alterations in hepatic gene expression. Whether or not the microdomains of milk SM and cholesterol revealed in the MFGM have some special functions still remains unknown. The roles of the physical structure and single components of the MFGM need additional studies to be understood.

## Conclusion

This overview has documented the current state of the art in relation to the influence of technological processes on the organization of lipids in various dairy products. The relationship between the composition, the structure of lipids and their functions is not fully understood. Recent advanced microscopy studies have permitted to gain important knowledge in situ in complex food products, and their development will progress in the next years. Significant knowledge gaps remain in areas including (i) the role of the organization of fat and microstructure of the acid gel formed in the stomach on the mechanisms of digestion and (ii) the specific role of the MFGM around milk fat globules vs processed lipid droplets even if they are coated by a monolayer of polar lipids. The biofunctionality of the MFGM is not simply a sum of those properties from individual molecules but could result from the specific organization and structure of the polar lipids, cholesterol and membrane proteins. Further nutritional studies are required to investigate the role of milk fat structure on its digestibility and metabolic fate of fatty acids. The role played by specific lipids (e.g. milk sphingomyelin) on the physiology of the intestine and on the microbiota will also improve the knowledge and participate in the improvement of dairy products for beneficial health impacts in infants and adults.
